# Effect of Powder-to-Liquid Ratio on pH, Calcium Ion Release, and Solubility Behaviors of Endodontic Bioceramics: An In Vitro Study

**DOI:** 10.3390/jfb17050220

**Published:** 2026-05-02

**Authors:** Asuka Aka, Takashi Matsuura, Atsutoshi Yoshimura

**Affiliations:** Department of Periodontology and Endodontology, Nagasaki University Graduate School of Biomedical Sciences, Nagasaki City 852-8588, Japan; a-aka@nagasaki-u.ac.jp (A.A.); ayoshi@nagasaki-u.ac.jp (A.Y.)

**Keywords:** bioceramics, bioceramic sealer, physicochemical properties, MTA Flow White, MTA Repair HP, Nishika Canal Sealer BG multi

## Abstract

This study investigated the physicochemical properties of three endodontic bioceramics: MTA Flow White (F), MTA Repair HP (HP), and Nishika Canal Sealer BG multi (BG). Disc-shaped samples were immersed in deionized water for 28 days to analyze pH, calcium ion concentration, mass change, and water sorption. Additionally, the effect of varying powder-to-liquid (or paste) ratios was investigated for F and BG. All samples exhibited mass loss due to surface degradation. Results showed that F exhibited the highest alkalinity (pH 10.8–11.3) and significantly greater calcium ion release (173.3–523.3 ppm) than other materials (*p* < 0.05). HP showed moderate alkalinity (pH 10.4–10.7) with lower calcium release (43.3–66.3 ppm), while BG exhibited the lowest alkalinity (pH 9.3–9.4). Regarding the effect of consistency, variations in the powder-to-liquid (or paste) ratio significantly influenced the physical stability of F and BG—notably shifting F from mass loss to mass gain—but did not significantly affect their pH or calcium ion release kinetics (*p* > 0.05). Consequently, both null hypotheses were rejected, as significant differences were observed among the materials, and consistency significantly affected mass change and water sorption but not alkalinity or ion release.

## 1. Introduction

The advent of mineral trioxide aggregate (MTA) in the early 1990s fundamentally transformed endodontic treatment protocols [1]. ProRoot MTA was the first calcium silicate-based cement approved for clinical use and is currently regarded as the “gold standard” for various endodontic applications, including root-end filling, perforation repair, and regenerative procedures [2,3,4,5]. Its widespread adoption is largely attributed to its favorable biocompatibility and ability to induce hard-tissue formation [3,6,7,8]. Nonetheless, the material is limited by challenging handling characteristics and a tendency for washout [3,4]. To overcome these persistent clinical limitations, further developments in bioceramic materials have emerged [3,4,9].

MTA Repair HP (HP; Angelus, Londrina, Brazil) was engineered to enhance clinical manipulation by adding a plasticizer to its liquid component [10]. Furthermore, the inclusion of calcium tungstate as a radiopacifying agent ensures adequate radiopacity without the risk of tooth staining [11,12,13]. Regarding its physical performance, HP exhibits a favorable push-out bond strength compared with traditional formulations [12]. While these clinical handling and adhesion characteristics are enhanced, the essential properties—including setting time, radiopacity, solubility, and water absorption—remain consistent with the established standards of conventional MTA [12,14].

MTA Flow White (F; Ultradent Products Inc., South Jordan, UT, USA) is a Portland cement-derived bioceramic consisting of tricalcium and dicalcium silicate powders mixed with a water-based gel. A key feature of this material is its adjustable powder-to-gel ratio, which allows clinicians to adjust the consistency from a thin fluid to a putty-like state depending on the clinical need. When prepared in a thin consistency, it can be efficiently delivered into the root canal system using 29-G NaviTip needles (Ultradent Products Inc., South Jordan, UT, USA) [3,15]. While its physicochemical, biological, and antimicrobial performance is equivalent to that of ProRoot MTA, it offers more favorable esthetic outcomes [16]. Specifically, tooth discoloration issues seen in the original MTA Flow were addressed by adopting a white powder base and replacing bismuth oxide with tantalum oxide as the radiopacifying agent [17].

In Japan, “dental root canal filling materials” are defined by regulation as materials that “set without the assistance of moisture and are used for the permanent sealing of root canals, regardless of the concomitant use of root canal filling points.” This specific regulatory framework has influenced the development of unique bioceramic sealers in the Japanese market, such as Nishika Canal Sealer BG multi (BG; Nippon Shika Yakuhin Co., Ltd., Yamaguchi, Japan). Unlike many international bioceramic sealers that are hydraulic (relying on environmental moisture to set), BG uses a dual-paste system in which the reaction between fatty acids in Paste A and magnesium oxide in Paste B achieves setting without external moisture. A distinct clinical advantage of BG is its versatility in handling; by incorporating a supplementary powder composed of calcium silicate glass and calcium hydroxide, clinicians can adjust its consistency from an injectable paste to a putty-like state. The material utilizes bismuth subcarbonate to ensure adequate radiopacity [18]. Furthermore, BG is characterized by favorable physicochemical stability, biocompatibility, and sealing efficacy, while also remaining removable during retreatment [19,20,21].

The biological and physicochemical characteristics of endodontic bioceramics are largely determined by the ions released during and after setting, as well as by subsequent pH shifts in the local environment [22]. Typically, the hydration of calcium silicate-based materials produces highly alkaline calcium hydroxide. Although this high alkalinity and the liberation of calcium ions are beneficial for antimicrobial effects and the promotion of hard-tissue formation [23], adverse effects, such as extreme pH levels, material dissolution (mass loss), or high water sorption, may lead to tissue irritation or negatively impact the sealing integrity and dimensional stability of the material [24].

While we have previously evaluated the biological characteristics of these materials, comprehensive evaluations of their properties under varying consistencies remain limited [25]. Both F and BG are engineered with adjustable formulations to meet diverse clinical needs—F through its powder-to-gel ratio and BG via the addition of a supplementary powder. However, there is currently a critical lack of clinical standardization regarding the optimal mixing ratios for specific endodontic procedures. Although manufacturers provide guidelines for adjustment, the impact of these variations on long-term physicochemical stability remains insufficiently characterized. This study aims to address this clinical gap by providing a systematic evaluation of how consistency shifts influence the pH, calcium ion concentration, water sorption, and mass change of HP, F, and BG (Table 1). Furthermore, this study specifically investigated the effect of the powder-to-liquid (or paste) ratio on the pH, calcium ion concentration in the eluate, water sorption, and mass change in F and BG to provide further insight into the clinical preparation of these materials. The null hypotheses investigated in this study were that there would be no statistically significant differences among F, HP, BG, and the negative control (NC) group in pH values, calcium ion release, mass change, and water sorption characteristics. Furthermore, it was hypothesized that variations in the powder-to-liquid (or paste) ratio for F and BG would not significantly affect their alkalinity, ion release kinetics, or physical stability.

## 2. Materials and Methods

**Figure 1:**
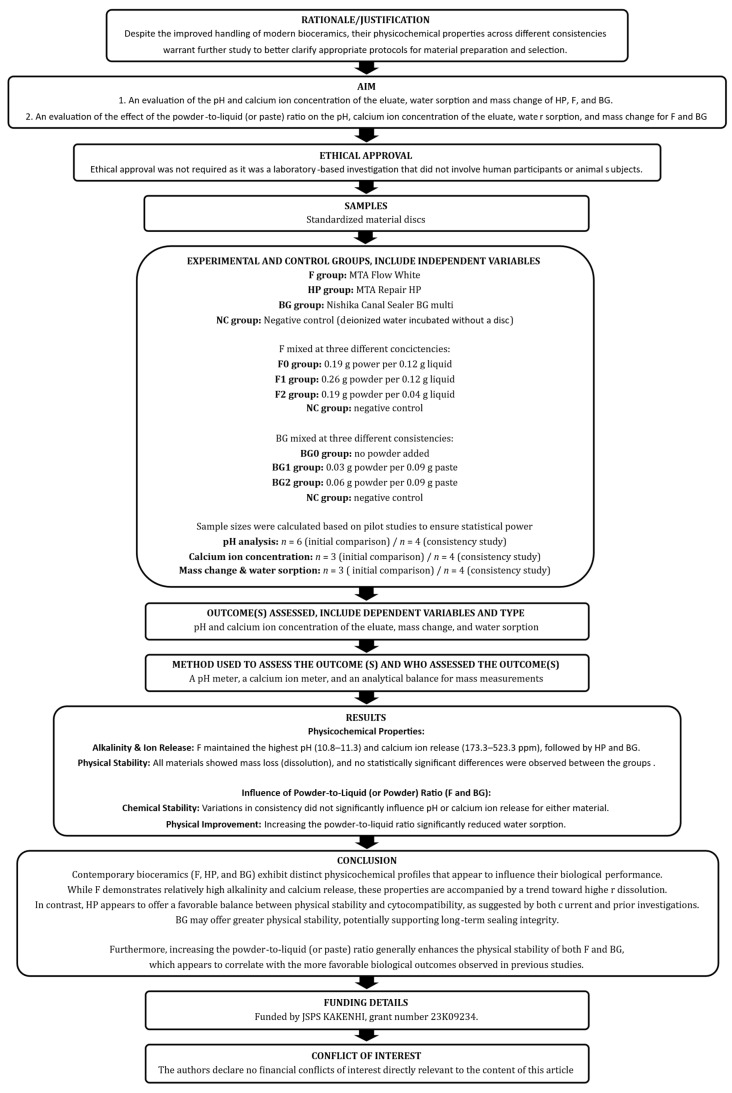
The PRILE 2021 flowchart of this study.

This laboratory investigation adhered to the Preferred Reporting Items for Laboratory studies in Endodontology (PRILE) 2021 guidelines [26,27]. Detailed compliance is illustrated in the PRILE 2021 flowchart (Figure 1) and the accompanying checklist (Table S1).

### 2.1. Bioceramic Disc Preparation

Three endodontic bioceramics (F, HP, and BG) were evaluated (Table 1). All materials were prepared inside a clean bench with sterile instruments according to the manufacturers’ instructions at a room temperature of 23 ± 2 °C, packed into molds (inner diameter = 8 mm, thickness = 1 mm), and incubated in a 5% CO_2_ incubator at 37 °C and saturated humidity (relative humidity ≥ 95% for 48 h to form standardized discs. F was prepared to have a soft consistency (powder-liquid ratio: 0.19 g powder per 0.12 g liquid). BG was mixed without adding powder.

### 2.2. Evaluation of pH Levels and Calcium Ion Concentration in the Eluate

Prepared discs were immersed in 500 μL of deionized water (Milli-Q; Millipore, Billerica, MA, USA) with an initial pH of approximately 7.0 in a 48-well plate. They were maintained in a humidified incubator (37 °C, 5% CO_2_, saturated humidity (relative humidity ≥ 95%) for 28 days. The immersion medium was replaced every 2 to 3 days until day 28, and the pH (*n* = 6) and calcium ion concentrations (*n* = 3) in the eluate were quantified on days 3, 5, 7, 10, 12, 14, 17, 19, 21, 24, 26, and 28 using a pH meter (S2K333, Toyorika, Tokyo, Japan) and a calcium ion meter (LAQUAtwin Ca-11; Horiba, Kyoto, Japan), respectively. These sample sizes were determined via power analysis based on preliminary pilot studies to ensure sufficient statistical power (see Section 2.6 for details). Deionized water (Milli-Q; Millipore, Billerica, MA, USA) with an initial pH of approximately 7.0, incubated without a disc, served as the NC. The pH of all samples was measured sequentially in the order of F, HP, BG, and NC.

### 2.3. Mass Change and Water Sorption

The initial mass (M_0_) of each disc was recorded using an analytical balance (Sartorius ED124S, Göttingen, Germany) immediately following a 48-h setting period. Subsequently, specimens were immersed in 500 μL of deionized water within a 48-well plate and maintained at 37 °C (5% CO_2_, saturated humidity (relative humidity ≥ 95%) for 28 days. The immersion medium was refreshed every 2–3 days. On day 28, discs were retrieved, gently blotted with filter paper to eliminate surface moisture, and weighed to obtain the final wet mass (M_f_) (*n* = 3). To determine the final dry mass (M_d_), the samples were returned to the incubator for an additional 48 h before final weighing. The percentage of mass change and water sorption were determined using Equations (1) and (2), respectively:

(1) $$Mass\;Change\left(\%\right)=\frac{M_{d}-M_{0}}{M_{0}}\times 100$$

(2) $$Water\;Sorption\left(\%\right)=\frac{M_{f}-M_{d}}{M_{d}}\times 100$$

### 2.4. Effects of the Powder-Liquid (Or Paste) Ratio of F or BG

F and BG were prepared at three different consistencies: F0 (thin: 0.19 g powder per 0.12 g liquid), F1 (thick: 0.26 g powder per 0.12 g liquid), and F2 (putty: 0.19 g powder per 0.04 g liquid); BG was also prepared at three different consistencies: BG0 (thin: no powder added), BG1 (thick: 0.03 g powder per 0.09 g paste), and BG2 (putty: 0.06 g powder per 0.09 g paste). These ratios were selected based on the manufacturers’ technical guidelines to represent the range of consistencies typically employed in clinical practice, from a thin fluid for syringe delivery to a putty-like state for filling procedures. Discs of F0-2 and BG0-2 were prepared, immersed in deionized water for 28 days, and the eluate was analyzed. The pH and calcium ion concentration of the eluate, and mass change/water sorption were measured on days 3, 5, 7, 10, 12, 14, 17, 19, 21, 24, 26, and 28, with four independent samples (*n* = 4) utilized for each experimental condition.

### 2.5. Statistical Analysis

For analysis of pH kinetics and calcium ion release, differences over time and between materials, and interaction between these factors were assessed using two-way repeated-measures ANOVA. The Bonferroni correction was applied to post hoc multiple comparisons to control the family-wise error rate. The level of statistical significance was set at α = 0.05.

To analyze mass change and water sorption, the Kruskal–Wallis test was performed to compare multiple groups. When significant differences were detected, Dunn’s test was used for post hoc pairwise comparisons. The level of statistical significance was set at α = 0.05. All data are presented as mean ± standard deviation. IBM SPSS Statistics (version 27.0; IBM Corp., Armonk, NY, USA) was used for all statistical analyses.

### 2.6. Sample Size

Sample sizes were determined via power analysis based on preliminary pilot studies (detailed in Figures S1–S6, Tables S2–S7). For all calculations, the power was set at 90%, and a 20% potential dropout rate was accounted for in the final enrollment.

For the comparisons among F, HP, and BG, sample sizes were calculated as follows:

- **pH:** Based on a predicted difference between F (10.5 [0.02]) and BG (9.3 [0.04]) groups observed in the pilot data (Figure S1, Table S2), six samples were enrolled per group (*α* = 0.05).
- **Calcium ion release:** Based on a difference between F (480.0 [0.0]) and NC (45.0 [1.4]) groups observed in the pilot data (Figure S2, Table S3), three samples were enrolled per group (*α* = 0.05).
- **Mass change:** Based on a difference between BG (−16.0 [0.2]) and HP (−6.0 [0.6]) groups observed in the pilot data (Figure S3, Table S4), three samples were enrolled per group (*α* = 0.05)

For the evaluation of powder-to-liquid (or paste) ratios of F or BG, sample sizes were determined as follows:

- **pH and Calcium ion release:** Based on differences between BG0 (pH: 9.1 [0.09]; Ca: 72.0 [8.7]) and NC (pH: 8.7 [0.10]; Ca: 0.0 [0.0]) groups observed in the pilot data (Figures S4 and S5, Tables S5 and S6), four samples were enrolled per group (*α* = 0.05).
- **Mass change and water sorption:** Based on the difference between F0 (87.2 [18.3]) and F2 (−12.0 [13.2]) groups observed in the pilot data (Figure S6, Table S7), as well as BG0 (32.5 [21.6]) and BG2 (−41.2 [8.1]) groups, four samples were used for each comparison (*α* = 0.05).

## 3. Results

### 3.1. pH Kinetics

**Figure 2:**
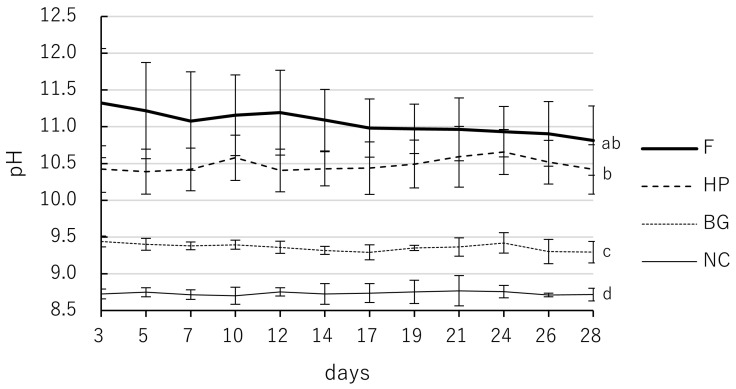
Time-dependent pH changes of F, HP, and BG over 28 days. Samples were immersed in deionized water, and the eluate was analyzed on days 3, 5, 7, 10, 12, 14, 17, 19, 21, 24, 26, and 28. F: MTA Flow White; HP: MTA Repair HP; BG: Nishika Canal Sealer BG multi; NC: negative control. Values indicate mean, and error bars represent standard deviation, and solid, thin solid, dashed, and dotted lines represent different materials as indicated in the legend. All tested materials exhibited significantly higher values than the NC group (*p* < 0.05). Values with different superscript letters (a, b, c, d) within the same column indicate statistically significant differences between the materials at day 28 (*p* < 0.05).

All tested materials significantly increased the pH of the eluate compared to the NC throughout the 28 days (Table 2, Figure 2). Two-way repeated-measures ANOVA revealed that both material type and time had significant effects on pH values (*p* < 0.05; specifically, *p* < 0.001), with a significant interaction between these factors (*p* < 0.05; specifically, *p* < 0.001). F maintained the significantly highest alkalinity among all groups at most time points until day 19 (*p* < 0.05). From day 21 to day 28, no statistically significant difference was observed between F and HP (*p* > 0.05), although F still exhibited numerically higher mean values. HP exhibited moderate alkalinity, maintaining pH values between 10.4 and 10.7. BG showed the lowest pH values (9.3–9.4) among the bioceramic materials, although they were still significantly higher than those of the NC group (8.7–8.8). The differences between the four groups generally followed the order of F > HP > BG > NC, with significant differences (*p* < 0.05) observed at most intervals, except for the comparison between F and HP in the final week.

### 3.2. Calcium Ion Release

**Figure 3:**
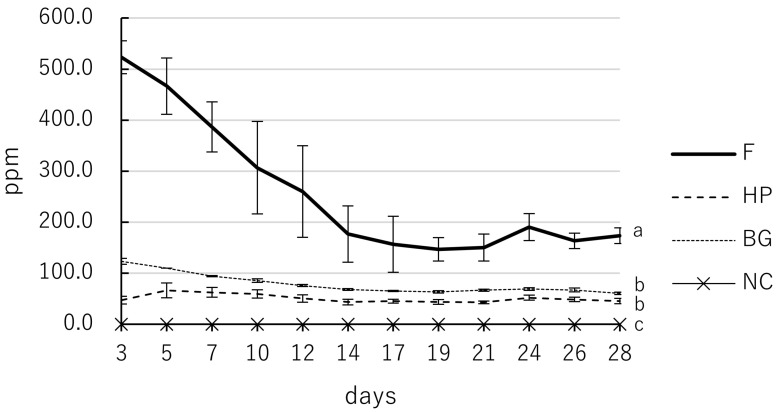
Calcium ion concentration kinetics of F, HP, and BG over 28 days. Samples were immersed in deionized water, and the eluate was analyzed on days 3, 5, 7, 10, 12, 14, 17, 19, 21, 24, 26, and 28. F: MTA Flow White; HP: MTA Repair HP; BG: Nishika Canal Sealer BG multi; NC: negative control. Values indicate mean, and error bars represent standard deviation, and solid, thin solid, dashed, and dotted lines represent different materials as indicated in the legend. All tested materials exhibited significantly higher values than the NC group (*p* < 0.05). Values with different superscript letters (a, b, c) within the same column indicate statistically significant differences between the materials at day 28 (*p* < 0.05).

The calcium ion concentration in the eluate varied significantly with both material type and immersion duration (*p* < 0.05; specifically, *p* < 0.001), with a significant interaction between these factors (*p* < 0.05; specifically, *p* < 0.001; Table 3, Figure 3). F demonstrated a robust and significantly higher release of calcium ions compared to all other materials, peaking at early intervals and maintaining high levels ranging from 173.3 to 523.3 ppm throughout the 28-day study (*p* < 0.05). BG exhibited intermediate release levels (60.3–123.3 ppm), which were significantly higher than those of HP during the initial 12 days (*p* < 0.05). In contrast, HP exhibited the most stable and gradual release pattern, with concentrations between 43.3 and 66.3 ppm, and no significant fluctuations over the 28 days. The NC group consistently showed 0.0 ppm calcium ion release, which was significantly lower than that of all tested bioceramic materials (*p* < 0.05).

### 3.3. Mass Change and Water Sorption

**Figure 4:**
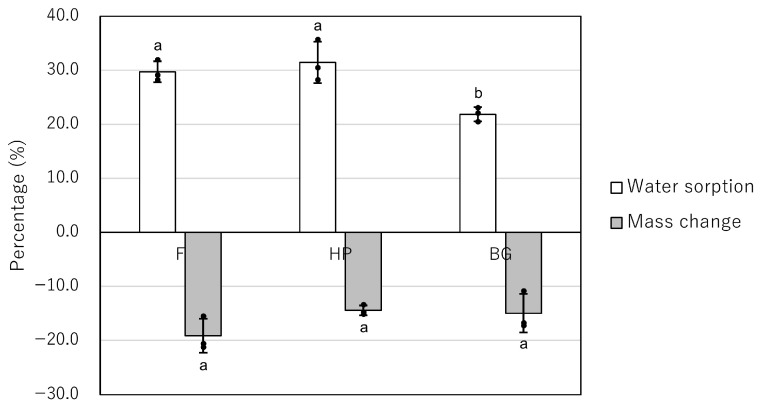
Mass change (%) and water sorption (%) of F, HP, and BG after 28 days of immersion. F: MTA Flow White; HP: MTA Repair HP; BG: Nishika Canal Sealer BG multi. Values indicate the mean. Positive values represent weight gain, whereas negative values represent material dissolution (mass loss). Individual data points are shown as black circles, and bars represent the mean value with error bars indicating standard deviation. Different indicators (a, b) above the bars indicate statistically significant differences among the groups (*p* < 0.05).

All materials exhibited a negative mass change, indicating dissolution during the 28-day immersion period (Table 4, Figure 4). While F showed the greatest mean mass loss (−19.2%), followed by BG (−15.0%) and HP (−14.5%), no statistically significant differences were observed between the groups (*p* > 0.05). In contrast, water sorption differed significantly among the materials (*p* < 0.05). Post hoc analysis using Dunn’s test indicated that BG exhibited significantly lower water sorption (21.8 ± 1.3%) compared to both F (29.7 ± 1.9%, *p* = 0.04) and HP (31.4 ± 3.8%, *p* = 0.04). There was no statistical difference in water sorption between F and HP (*p* > 0.05).

### 3.4. pH Kinetics of F and BG at Different Consistencies

**Figure 5:**
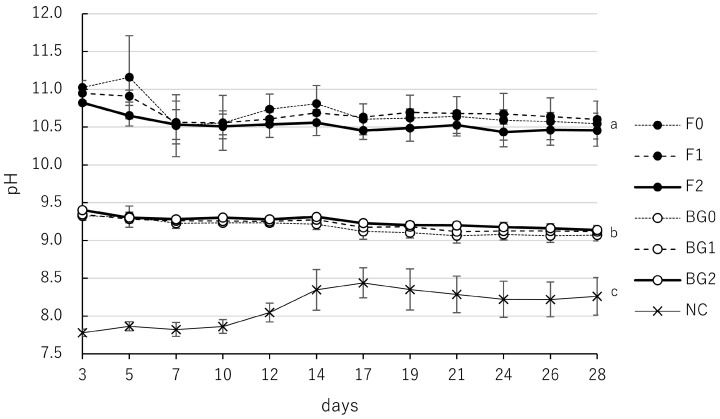
Time-dependent pH changes of F and BG at different consistencies over 28 days. Samples were immersed in deionized water, and the eluate was analyzed on days 3, 5, 7, 10, 12, 14, 17, 19, 21, 24, 26, and 28. Consistencies for F (black circles): F0 (thin; dotted line), F1 (thick; dashed line), and F2 (putty; solid line); Consistencies for BG (open circles): BG0 (thin; dotted line), BG1 (thick; dashed line), and BG2 (putty; solid line). Detailed mixing ratios are provided in the Materials and Methods Section; NC represents the negative control (crosses with a thin solid line). Values indicate mean, and error bars represent standard deviation. All tested materials exhibited significantly higher values than the NC group (*p* < 0.05). Values with different superscript letters (a, b, c) within the same column indicate statistically significant differences between the groups at day 28 (*p* < 0.05).

The pH values of the eluate from F and BG prepared at three different consistencies (F0–F2 and BG0–BG2) were monitored over 28 days (Table 5, Figure 5). All experimental groups for both materials maintained significantly higher alkalinity than the NC group throughout the study. pH values of F showed no statistically significant differences among F0, F1, and F2, with all groups sustaining high alkalinity levels ranging from 10.4 to 11.2. Similarly, the pH values for the BG groups (BG0, BG1, and BG2) showed no significant differences across the varying consistencies, remaining stable within a range of 9.1 to 9.4. Two-way repeated-measures ANOVA and post hoc multiple comparisons confirmed that while material type and time had significant effects, the specific powder-to-liquid (or paste) ratio did not significantly affect alkalinity for either material.

### 3.5. Calcium Ion Release of F and BG at Different Consistencies

**Figure 6:**
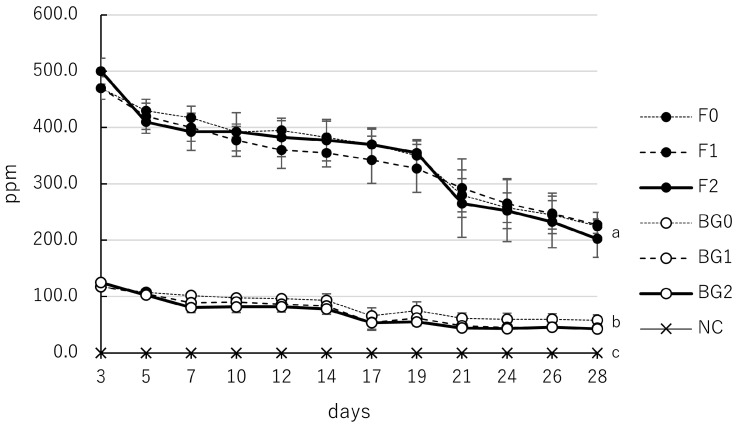
Calcium ion release (ppm) from F and BG at different consistencies over 28 days. Samples were immersed in deionized water, and the eluate was analyzed on days 3, 5, 7, 10, 12, 14, 17, 19, 21, 24, 26, and 28. Consistencies for F (black circles): F0 (thin; dotted line), F1 (thick; dashed line), and F2 (putty; solid line); Consistencies for BG (open circles): BG0 (thin; dotted line), BG1 (thick; dashed line), and BG2 (putty; solid line). Detailed mixing ratios are provided in the Materials and Methods Section; NC represents the negative control (crosses with a thin solid line). Values indicate mean, and error bars represent standard deviation. All tested materials exhibited significantly higher values than the NC group (*p* < 0.05). Values with different superscript letters (a, b, c) within the same column indicate statistically significant differences between the groups at day 28 (*p* < 0.05).

The calcium ion release kinetics showed that all tested groups for both F and BG exhibited a gradual decrease in ion concentration over 28 days (Table 6, Figure 6). F maintained high calcium ion release across all consistencies, with values starting at 470.0–500.0 ppm on day 3 and declining to 202.5–227.5 ppm by day 28. Although F2 recorded the highest initial concentration on day 3, the overall release patterns and the rate of decline remained generally similar across the three consistencies (F0, F1, and F2), with no statistically significant differences observed between them at any time point (*p* > 0.05). For BG, all groups (BG0, BG1, and BG2) also demonstrated a reduction in calcium ion release over time, with concentrations decreasing from 117.5–125.0 ppm on day 3 to 42.5–58.0 ppm by day 28. All BG consistencies maintained significantly higher calcium ion release than the NC group throughout the study (*p* < 0.05), yet no significant differences were found between the different BG consistencies themselves (*p* > 0.05).

### 3.6. Mass Change and Water Sorption of F and BG at Different Consistencies

**Figure 7:**
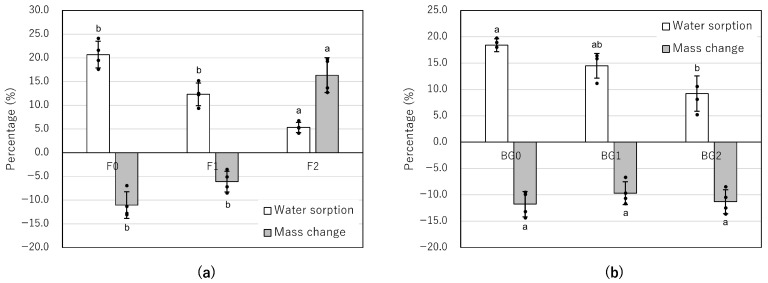
Mass change (%) and water sorption (%) of F and BG at different consistencies after 28 days of immersion in deionized water. F: MTA Flow White; BG: Nishika Canal Sealer BG multi. (**a**) F mixed at three consistencies—F0 (0.19 g powder per 0.12 g liquid), F1 (0.26 g powder per 0.12 g liquid), and F2 (0.19 g powder per 0.04 g liquid); (**b**) BG mixed at three consistencies—BG0 (no powder added), BG1 (0.03 g powder per 0.09 g paste), and BG2 (0.06 g powder per 0.09 g paste). Values indicate the mean. Positive values indicate weight gain, while negative values indicate material dissolution (mass loss). Individual data points are shown as black circles, and bars represent the mean value with error bars indicating standard deviation. Different indicators (a, b) above the bars indicate statistically significant differences among consistencies within each property (*p* < 0.05).

The mass change and water sorption of F and BG prepared at varying powder-to-liquid (or paste) ratios are presented in Table 7 and Figure 7. For the F groups, the Kruskal–Wallis test showed significant differences in both mass change (*p* = 0.005) and water sorption (*p* = 0.004). Increasing the powder-to-liquid ratio led to a distinct shift from material dissolution (mass loss) to substantial mass gain in the F2 group (Table 7, Figure 7a). Specifically, F2 exhibited a mean mass increase of 16.3 (3.7)%, while F0 and F1 continued to show mass loss (−11.1% and −6.1%, respectively). Post hoc Dunn’s test revealed that F2 had a significantly different mass-change profile, characterized by mass gain, compared with the thinner consistencies F0 (*p* = 0.008) and F1 (*p* = 0.041) (Table 7, Figure 7a). In contrast, water sorption in F significantly decreased as the powder-to-liquid ratio increased, with F2 showing the lowest values (*p* < 0.05).

The BG groups demonstrated greater physical stability than the F groups, with minimal mass fluctuations across all tested consistencies (Table 7, Figure 7b). The mass change for all BG variations remained stable, ranging from −11.8 (2.4)% for BG0 to −9.7 (2.2)% for BG1 (and −11.3 [2.3]% for BG2). No significant difference in mass change was observed among the three consistencies (*p* > 0.05), indicating that these materials experienced consistent but minor dissolution regardless of the powder-to-liquid ratio. As with the F groups, water sorption was significantly affected by consistency (*p* < 0.05). Water sorption in the BG samples decreased with increasing powder content. BG2 exhibited significantly lower water sorption (9.2 [3.4]%) compared to BG0 (18.4 [1.2]%) (*p* = 0.008).

## 4. Discussion

This study characterized the physicochemical properties of three endodontic bioceramics and demonstrated that increasing the powder-to-liquid (paste) ratio significantly enhances their physical stability. Traditionally, endodontic sealers are compared with one another [28,29,30]. Recently, however, bioceramic sealers such as BG have been increasingly used in repair procedures, including perforation repair and apical plug placement—applications traditionally reserved for MTA. This shift necessitates a direct comparison between bioceramic sealers and established repair materials such as MTA. Consequently, the present study compared BG with HP and F, utilizing a disc-based model commonly used in in vitro MTA research. This methodological approach revealed that BG, despite its primary classification as a sealer, exhibits physical stability comparable to that of conventional repair materials. In this study, BG exhibited the lowest pH values (9.3–9.4) and significantly lower water sorption (21.8%) compared to F (29.7%) and HP (31.4%), which may be favorable for long-term dimensional stability in bulk applications. Furthermore, while F showed a substantial transition from material dissolution to expansion depending on the powder-to-liquid ratio, the mass change for all BG variations remained relatively stable. This may suggest that the fatty acid-based matrix of BG contributes to a stable physical profile under the conditions of this study, even when the material is prepared in higher consistencies.

### 4.1. The Relationship Between Decreasing Calcium Ion Release and Sustained Alkalinity

In this study, F maintained a significantly high alkalinity (pH 10.8–11.3) throughout the 28 days despite a reduction in calcium ion concentration (from 523.3 ppm on day 3 to 173.3 ppm on day 28). This observation can be explained by the definition of pH, which is the negative logarithm of H^+^ ion activity. The relationship between pH and the concentration of OH^−^ ions is expressed by the following Equations (3) and (4) [31]:

(3) pOH=14−pH

(4) $$[{OH}^{-}]=10^{-pOH}$$

Therefore, a substantial reduction in OH^−^ release—even by half—results in only a minor shift in the numerical pH value. For instance, while the pH of F shifted by only 0.5, from 11.3 at day 3 to 10.8 at day 28, the calculated concentration of OH^−^ ions actually decreased by approximately 68.5%, dropping from 34.0 ppm to 10.7 ppm. This rate of reduction in OH^−^ concentration is highly consistent with the decrease observed in calcium ion release, which dropped by approximately 66.9% during the same period. This correlation in reduction rates (calcium ion release (66.9%) and the calculated OH^−^ concentration (68.5%)) suggests that the release of these ions is likely governed by the dissociation of calcium hydroxide.

This trend of decreasing ion release warrants further consideration of the underlying physical mechanisms. The observed reduction in calcium ion release over the 28 days (Table 3 and Table 6) does not appear to be attributed solely to the reduction in material mass. It might be suggested that the ongoing hydration of the silicate particles and the subsequent formation of a mineralized surface layer contribute to this trend. The accumulation of precipitates, such as calcium carbonate, could potentially act as a physical barrier that restricts the outward diffusion of ions into the deionized water. Furthermore, the periodic replacement of the immersion medium (every 2–3 days) may account for the observed fluctuations in ion concentration, as the restoration of the concentration gradient appears to facilitate the elution of ions from the material–liquid interface periodically.

### 4.2. The Relationship Between Physicochemical Properties and Cytocompatibility

In the present study, the physicochemical properties of endodontic bioceramics, including F, HP, and BG, were evaluated at specific time points—days 3, 5, 7, 10, 12, 14, 17, 19, 21, 24, 26, and 28—to align precisely with the medium-exchange schedule used in our previous investigation of cytocompatibility [25]. This approach may enable a more direct correlation between the material’s ion release kinetics and its biological effects on human periodontal ligament-derived cells (hPDLCs) over 28 days. Consequently, several significant insights regarding the biological behavior of these bioceramics can be drawn.

HP was reported to demonstrate a more favorable biological profile [32]. In our previous study, an evaluation using a 3-[4,5-dimethylthiazol-2-yl]-2,5-diphenyltetrazolium bromide (MTT) assay revealed that HP exhibited high cytocompatibility (absorbance 2.87 [0.35] at day 28), comparable to the NC (2.64 [0.32]) [25]. Moderate alkalinity (pH 10.4–10.7) and a stable, gradual calcium ion release pattern observed in this study are considered among the factors contributing to this favorable biological performance. Furthermore, HP exhibited the lowest mass change among the bioceramics (−14.5%), suggesting that its physical stability prevents the sudden chemical shifts that trigger cellular stress. This physical stability is also considered a factor contributing to its favorable biological performance.

In contrast, F maintained the highest alkalinity (pH 10.8–11.3) and the greatest calcium ion release (173.3–523.3 ppm), consistent with previous studies [15]. However, F exhibited the lowest cellular metabolic activity (absorbance 0.23 [0.67]) among the tested materials [25]. This suggests that while high pH and calcium release are beneficial for antimicrobial effects and biomineralization, they may create an excessively harsh microenvironment for hPDLCs in a closed in vitro system [33]. The high mass loss of F (−19.2%) further indicates that rapid dissolution may have led to an accumulation of leached components at concentrations exceeding the cells’ physiological tolerance. These data indicated that initial and sustained chemical shifts, such as those associated with extreme pH and high mass loss, may influence the longevity of cell viability in vitro.

### 4.3. Comparison of HP Physicochemical Properties

In a study by Ferreira et al., the mass change of HP was reported as 2.46 (0.9)% [14]. This minimal change suggests that mass gain from hydration reactions may partially offset material dissolution under certain conditions. In contrast, the present study observed a mass decrease of −14.5 (0.9)%. This discrepancy could be attributed to the difference in experimental protocols. Specifically, the frequent replacement of the immersion medium in our study might have prevented the solution from reaching saturation, thereby facilitating the continuous elution of material components over the 28 days. Furthermore, the water sorption value in this study (31.4 (3.8)%) was notably different from the 10.84 (1.7)% reported by Ferreira et al. [14]. This variation might be explained by the following factors. First, frequent liquid exchange maintains a concentration gradient that may enhance the osmotic drive, potentially increasing water penetration compared to static immersion models. Second, our 28-day evaluation, compared with the 7-day interval used in prior research, may have allowed for greater moisture penetration into the material’s core. This extended timeframe may have triggered delayed hydration of unreacted particles, which might have been further highlighted by the dynamic ion-exchange process observed in this study.

### 4.4. The Influence of the Powder-to-Liquid Ratio of F on Biological Outcomes

In contrast to premixed formulations such as Bio-C Repair, which were evaluated in our previous investigation, materials such as F and BG allow clinician-guided adjustments [34]. Our findings demonstrate that these variations in consistency not only alter handling characteristics but also significantly influence mass change and water sorption, potentially affecting the biological microenvironment.

In the present study, variations in the powder-to-liquid ratio of F did not significantly affect its alkalinity (Table 5, Figure 5) or the kinetics of calcium ion release (Table 6, Figure 6). However, physical stability was notably affected: increasing the powder content (higher consistency) reduced mass loss for F1 and led to mass gain for F2. Specifically, while F0 exhibited a mean mass loss of −11.1%, F1 showed a reduced mass loss of −6.1%, and F2 demonstrated a mean mass increase of 16.3%. (Table 7, Figure 7a). This expansive behavior in the F2 group, accompanied by a reduction in water sorption (from 20.7% in F0 to 5.3% in F2), appears to correlate with the biological outcomes observed in our previous investigation. The MTT assay with hPDLCs revealed that cellular metabolic activity at day 28 increased with higher consistency, following the order: F2 (absorbance 2.18 [1.09]) > F1 (1.44 [1.20]) > F0 (1.02 [1.06]) [25]. This biological improvement correlates with the mass change data: the mitigation of material dissolution in F1, and the distinct mass gain in F2. The improved cytocompatibility in higher-consistency groups may be attributed to two distinct mechanisms related to their physical stability. First, for both F1 and F2, the higher powder-to-liquid ratio limited water influx and the subsequent efflux of unreacted components at potentially toxic concentrations. This is supported by the lower mass loss in F1 (−6.1%) compared to F0 (−11.1%), which contributed to a more stable, less chemically stress-inducing microenvironment. Second, the significant mass gain observed specifically in F2 (16.3%) likely reflects delayed hydration of previously unreacted powder particles and the accumulation of mineral precipitates, such as calcium carbonate or hydroxyapatite, on the material’s surface. This process not only increases the material’s weight but also may provide a more bioactive and stable substrate for cell attachment, further supporting the highest metabolic activity observed in the F2 group. Furthermore, the shorter setting time associated with higher consistency might have further supported cell viability [35].

The initial pH of F (thick consistency) in a previous study was reported as approximately 10.1, slightly lower than the value observed in the present study (pH 10.8–11.3) [16]. This discrepancy is likely attributable to their study’s use of elution in a cell culture medium with buffering capacity. This suggests that under conditions more closely resembling the actual biological environment, F may provide the alkaline environment necessary for treatment while moderately suppressing tissue irritation caused by extreme alkalinity. Furthermore, the same study reported that hardened F exhibited no cytotoxicity and demonstrated favorable biocompatibility. In contrast, our previous investigation showed that F had relatively lower cellular metabolic activity than the other materials used in this study [16,25]. Potential reasons for this difference in biological outcomes include variations in material consistency and elution conditions. While Tušas et al. reported good biocompatibility with a thick consistency (3:2), our previous study used a “soft” consistency with higher fluidity. As inferred from the present study, increasing the consistency of F1 or F2 may improve physical stability and enhance the chemical microenvironment for cells. These findings suggest that selecting an appropriate level of consistency may be crucial for enabling the material to fully exert its inherent biocompatibility, as demonstrated in prior research.

### 4.5. The Influence of Powder-to-Paste Ratio of BG on Biological Outcomes

In the present study, BG exhibited high physical stability, with consistent, minimal mass change across all consistencies, despite a trend toward decreased water sorption as the powder-to-paste ratio increased (Table 7, Figure 7b). Unlike F, which showed a substantial shift from dissolution to expansion, the mass change for all BG variations remained stable within a narrow range of −9.7% to −11.8%. This indicates that BG undergoes consistent, minor, and controlled dissolution regardless of its consistency. This stability may be attributed to its dual-paste setting mechanism, involving a reaction between fatty acids in Paste A and magnesium oxide in Paste B. This fatty acid-based matrix likely provides a more hydrophobic environment than the water-based gel of F, thereby sequestering its chemical constituents and limiting material dissolution even after water absorption. These findings suggest that such physical characteristics may be one of the factors explaining the favorable biological profiles reported in our previous study [25]. While the rapid dissolution and high mass loss of F potentially led to the accumulation of leaching components that inhibited cellular metabolic activity, the stable profile of BG suggests reduced chemical stress on hPDLCs. Furthermore, the observation that higher consistency (BG2) improved cellular absorbance relative to the group with no powder added (BG0) correlates with reduced water sorption. By potentially limiting the influx of water into the material and the subsequent efflux of unreacted components, a higher powder-to-paste ratio may promote a more controlled, gradual ion exchange at the material–cell interface, contributing to the formation of a stable microenvironment conducive to cell viability. Furthermore, these chemical characteristics may also be one of the factors explaining the favorable biological profiles reported in our previous study [25].

### 4.6. Comparison of Ion Release Kinetics of BG with Previous Literature

A comparison of BG’s calcium ion release behavior in this study with that reported in [19] revealed a significant numerical discrepancy. While the previous study reported a peak calcium ion release of approximately 9.31 mg/L for BG (Day 7), the present study recorded approximately 123.3 ppm (mg/L) on Day 3, roughly 13 times higher. This discrepancy may be largely explained by differences in surface area-to-volume (SA/V) ratios across the experimental designs. The previous study used tubes with an inner diameter of 2 mm, in which only the cross-sectional area of the opening—approximately 3.14 mm^2^—was in contact with the immersion medium. In contrast, the present study employed discs (diameter 8 mm, thickness 1 mm) with all surfaces exposed, resulting in a total surface area of approximately 75.3 mm^2^ (50.2 mm^2^ face + 25.1 mm^2^ lateral edge). Furthermore, the volume of the immersion medium differed substantially: 10 mL in the previous study compared to a minimal 0.5 mL in the current study. Based on the SA/V ratio, the previous study’s ratio was approximately 0.31 mm^−1^, whereas the present study’s ratio was approximately 150.6 mm^−1^, representing a more than 400-fold increase in sample surface area per unit volume. In such a restricted environment, eluted ions are highly concentrated, which likely accounts for the elevated concentrations detected in ppm. Notably, despite this environment conducive to calcium ion concentration, the pH levels of BG in this study remained remarkably stable at 9.3–9.4 throughout the 28-day period, which was lower than the peak pH of 10.12 observed in previous research [19]. While calcium ions and OH^−^ ions typically correlate via Ca(OH)_2_ dissociation, the suppressed alkalinity in BG may be attributed to the neutralizing effect of fatty acid components. These findings cautiously suggest that the unique chemical profile of BG—providing a sufficient calcium supply while maintaining a moderate pH—may be a key factor in its favorable cytocompatibility, supporting the viability of hPDLCs.

### 4.7. Study Limitations

Several limitations should be acknowledged when evaluating the findings of this research. First, it should be noted that the sample size for some physical and chemical assessments was *n* = 3 for each group. Although these numbers were derived from power calculations aimed at detecting significant differences, the relatively small sample size suggests that the results should be interpreted with caution regarding their universal applicability. Second, this investigation was conducted under controlled in vitro conditions, which do not fully replicate the complex biological environment encountered in clinical practice. Additionally, using deionized water as the immersion medium, rather than a simulated body fluid, may artificially elevate pH and ion release values due to its lack of buffering capacity found in clinical environments. Furthermore, the pH values recorded for the NC group were higher than theoretically expected for pure water. This may be attributed to the low ionic strength of deionized water, which makes it highly susceptible to minor carry-over from the preceding alkaline samples during sequential measurement. While this represents a technical limitation in capturing the absolute baseline of the NC, the significant and consistent differences observed between the bioceramic materials and the NC group indicate that the comparative analysis of the materials’ alkalinity remains valid. Moreover, the current study focused on long-term kinetics with measurements starting from day 3. Consequently, the high-resolution behavior of pH and ion release during the first 72 h—the period when the most rapid changes occur—was not captured. Future studies with more frequent early-interval measurements are needed to fully characterize the initial setting reactions of these bioceramics. While the immersion volume (500 μL) was matched to our previous biological assays to ensure comparability, this relatively small volume may have influenced the ion release kinetics via saturation effects. Future studies with larger volumes or dynamic flow models may provide additional insights. Third, while this study evaluated the materials over 28 days, further observation is required to assess their long-term stability and behavior over several years of clinical function. Future in vivo studies and long-term assessments of sealing integrity are required to comprehensively evaluate the material’s performance in biological environments. Fourth, while this study hypothesized that the mass changes observed—specifically material dissolution in most groups and the mass gain in the F2 group—were driven by the efflux of components and the accumulation of mineral precipitates such as calcium carbonate or hydroxyapatite, these mechanisms were not morphologically or compositionally verified. The lack of direct surface characterization and structural analysis means these processes remain speculative. The progressive decrease in calcium ion concentration over 28 days may be explained by the maturation of the calcium silicate hydrate and the accumulation of precipitates, such as calcium carbonate, on the disc surface, which acts as a physical barrier to further ion diffusion. Furthermore, because this investigation used standardized, preset discs, it did not evaluate clinical handling characteristics, such as the ease of material disintegration or the degree of washout resistance during the initial setting phase. Additionally, the subtle fluctuations in ion levels observed at different intervals could be attributed to the periodic replacement of deionized water. This process repeatedly restores the concentration gradient, potentially causing transient increases in elution through micro-cracks or loosely bound surface precipitates. Future investigations employing scanning electron microscopy and energy-dispersive X-ray spectroscopy are essential to confirm these morphological changes, chemical composition, the precise role of surface mineralization in ion release kinetics, and the true clinical stability of these materials. Fifth, this study focused primarily on chemical and physical stability, such as pH kinetics, ion release, and mass change; however, other critical mechanical and clinical properties, including compressive strength and push-out bond strength, were not evaluated. Since the powder-to-liquid (paste) ratio and the resulting consistency may influence the mechanical integrity and adhesion of bioceramics to the dentin, these parameters should be addressed in future research to provide a more comprehensive understanding of their clinical performance.

### 4.8. Clinical Implications

The findings of this laboratory study suggest several potential clinical considerations for the application of these bioceramic materials. *F*: The observed shift from mass loss to mass gain in the putty-like consistency (F2) suggests that increasing the powder-to-liquid ratio may enhance physical stability. This characteristic could be advantageous for apical plug procedures where material dissolution and washout resistance are critical concerns. *BG*: This material exhibited stable mass change (within a range of approximately ± 2%) regardless of the powder-to-paste ratio. Such physical robustness across different consistencies may support its versatility as both a root canal sealer and a repair material, providing consistent behavior in various clinical scenarios. *HP*: The favorable biological profile identified in prior research appears to correlate with the moderate alkalinity and stable physical properties observed in the current study. These balanced characteristics might make it a reliable choice for clinical applications prioritizing tissue compatibility.

## 5. Conclusions

In summary, because significant variations were observed across all tested parameters with respect to both material selection and preparation consistency, the null hypotheses investigated in this study were rejected. This study demonstrates that contemporary endodontic bioceramics possess distinct physicochemical profiles that directly influence their biological behavior. F exhibited the highest alkalinity and calcium ion release, suggesting a greater potential for antimicrobial activity and biomineralization. However, these properties tended to coincide with higher material dissolution and less favorable cytocompatibility than those of the other materials in our prior study. HP maintained moderate alkalinity and a stable ion release pattern, which correlates with its more favorable cytocompatibility and physical stability observed in both current and prior studies. BG exhibited the lowest alkalinity but showed favorable physical stability, with significantly lower water sorption and a stable mass-change profile, making it a viable option for maintaining long-term sealing integrity. Increasing the powder-to-liquid (or paste) ratio consistently improved the physicochemical stability of the materials. In particular, the stepwise reduction in water sorption and the shift from mass loss to mass gain in the F groups suggest that increasing consistency, even to a moderate level (F1), enhances physical properties compared to the thin consistency (F0). These physical improvements, particularly in the higher-consistency groups, appear to be associated with the more favorable cytocompatibility observed for both F and BG in both current and prior investigations. Such findings may serve as a practical reference for clinicians when selecting and preparing bioceramic materials for specific endodontic procedures.

## Figures and Tables

**Table 1:** Materials used in this study. Reprinted from Ref. [25].

Materials	Manufactures	Composition
MTA Repair HP (HP)	Angelus (Londrina, Brazil)	powder: calcium silicate, calcium aluminate, calcium oxide, calcium tungstate liquid: purified water, plasticizer
MTA Flow White (F)	Ultradent Products (South Jordan, UT, USA)	powder: tricalcium silicate, dicalcium silicate, calcium sulfate, etc. gel: purified water, thickening agent
Nishika Canal Sealer BG multi (BG)	Nippon Shika Yakuhin (Yamaguchi, Japan)	paste A: fatty acids, bismuth bicarbonate, silicon dioxide paste B: magnesium oxide, purified water, calcium silicate glass, silicon dioxide, etc. powder: calcium silicate glass, calcium hydroxide

**Table 2:** pH values of endodontic bioceramics over 28 days.

Day	3	5	7	10	12	14	17	19	21	24	26	28
F	11.3 (0.7) ^a^	11.2 (0.7) ^a^	11.1 (0.7) ^ab^	11.2 (0.5) ^a^	11.2 (0.6) ^a^	11.1 (0.4) ^a^	11.0 (0.4) ^a^	11.0 (0.3) ^a^	11.0 (0.4) ^ab^	10.9 (0.3) ^ab^	10.9 (0.4) ^ab^	10.8 (0.5) ^ab^
HP	10.4 (0.3) ^b^	10.4 (0.3) ^b^	10.4 (0.3) ^b^	10.6 (0.3) ^b^	10.4 (0.3) ^b^	10.4 (0.2) ^b^	10.4 (0.4) ^b^	10.5 (0.3) ^b^	10.6 (0.4) ^b^	10.7 (0.3) ^b^	10.5 (0.3) ^b^	10.4 (0.3) ^b^
BG	9.4 (0.1) ^c^	9.4 (0.1) ^c^	9.4 (0.1) ^c^	9.4 (0.1) ^c^	9.4 (0.1) ^c^	9.3 (0.1) ^c^	9.3 (0.1) ^c^	9.4 (0.0) ^c^	9.4 (0.1) ^c^	9.4 (0.1) ^c^	9.3 (0.2) ^c^	9.3 (0.1) ^c^
NC	8.7 (0.1) ^d^	8.8 (0.1) ^d^	8.7 (0.1) ^d^	8.7 (0.1) ^d^	8.8 (0.1) ^d^	8.7 (0.1) ^d^	8.7 (0.1) ^d^	8.8 (0.2) ^d^	8.8 (0.2) ^d^	8.8 (0.1) ^d^	8.7 (0.0) ^d^	8.7 (0.1) ^d^

Values indicate mean (standard deviation). F: MTA Flow White; HP: MTA Repair HP; BG: Nishika Canal Sealer BG multi; NC: negative control. Values with different superscript letters (^a, b, c, d^) within the same column indicate statistically significant differences between the materials at that specific time point (*p* < 0.05 based on two-way repeated-measures ANOVA with Bonferroni correction).

**Table 3:** Calcium ion concentration over 28 days.

Day	3	5	7	10	12	14	17	19	21	24	26	28
F	523.3 (32.1) ^a^	466.7 (55.1) ^a^	386.7 (49.3) ^a^	306.7 (90.7) ^a^	260.0 (90.0) ^a^	176.7 (55.1) ^a^	156.7 (55.1) ^a^	146.7 (23.1) ^a^	150.0 (26.5) ^a^	190.0 (26.5) ^a^	163.3 (15.3) ^a^	173.3 (15.3) ^a^
HP	47.0 (7.5) ^c^	66.3 (14.6) ^c^	62.3 (9.7) ^c^	59.0 (8.2) ^c^	50.3 (7.4) ^c^	43.3 (5.5) ^b^	45.0 (3.6) ^c^	43.3 (4.7) ^c^	42.7 (2.5) ^c^	51.7 (4.9) ^c^	48.3 (4.5) ^c^	45.3 (5.1) ^b^
BG	123.3 (5.8) ^b^	110.0 (0.0) ^b^	94.0 (1.0) ^b^	85.3 (3.5) ^b^	75.7 (2.1) ^b^	68.0 (1.7) ^b^	65.0 (1.0) ^b^	63.3 (2.3) ^b^	66.7 (2.1) ^b^	69.0 (2.6) ^b^	67.0 (4.0) ^b^	60.3 (2.5) ^b^
NC	0.0 (0.0) ^d^	0.0 (0.0) ^d^	0.0 (0.0) ^d^	0.0 (0.0) ^d^	0.0 (0.0) ^d^	0.0 (0.0) ^c^	0.0 (0.0) ^d^	0.0 (0.0) ^d^	0.0 (0.0) ^d^	0.0 (0.0) ^d^	0.0 (0.0) ^d^	0.0 (0.0) ^c^

Values indicate mean (standard deviation). F: MTA Flow White; HP: MTA Repair HP; BG: Nishika Canal Sealer BG multi; NC: negative control. Values with different superscript letters (^a, b, c, d^) within the same column indicate statistically significant differences between the materials at that specific time point (*p* < 0.05 based on two-way repeated-measures ANOVA with Bonferroni correction).

**Table 4:** Mass change (%) and water sorption (%) of the tested materials after 28 days of immersion.

Material	Property	1	2	3	Mean ± SD
F	Mass change (%)	−20.6	−21.3	−15.5	−19.2 ± 3.1 ^a^
	Water sorption (%)	31.9	29.1	28.2	29.7 ± 1.9 ^a^
HP	Mass change (%)	−15.2	−14.8	−13.4	−14.5 ± 0.9 ^a^
	Water sorption (%)	35.7	28.2	30.5	31.4 ± 3.8 ^a^
BG	Mass change (%)	−10.9	−17.3	−16.8	−15.0 ± 3.6 ^a^
	Water sorption (%)	20.4	22.0	23.0	21.8 ± 1.3 ^b^

F: MTA Flow White; HP: MTA Repair HP; BG: Nishika Canal Sealer BG multi. Different lowercase letters (^a, b^) indicate significant differences among the groups within the same property (*p* < 0.05).

**Table 5:** pH values of F and BG at different consistencies over 28 days.

Day	3	5	7	10	12	14	17	19	21	24	26	28
F0	11.0 (0.1) ^a^	11.2 (0.5) ^a^	10.5 (0.4) ^a^	10.6 (0.4) ^a^	10.7 (0.2) ^a^	10.8 (0.2) ^a^	10.6 (0.2) ^a^	10.6 (0.3) ^a^	10.6 (0.3) ^a^	10.6 (0.4) ^a^	10.6 (0.3) ^a^	10.5 (0.3) ^a^
F1	11.0 (0.0) ^a^	10.9 (0.1) ^a^	10.6 (0.3) ^a^	10.6 (0.2) ^a^	10.6 (0.1) ^a^	10.7 (0.0) ^a^	10.6 (0.0) ^a^	10.7 (0.0) ^a^	10.7 (0.0) ^a^	10.7 (0.1) ^a^	10.6 (0.1) ^a^	10.6 (0.1) ^a^
F2	10.8 (0.0) ^a^	10.7 (0.1) ^a^	10.5 (0.2) ^a^	10.5 (0.2) ^a^	10.5 (0.2) ^a^	10.6 (0.2) ^a^	10.5 (0.1) ^a^	10.5 (0.2) ^a^	10.5 (0.1) ^a^	10.4 (0.1) ^a^	10.5 (0.1) ^a^	10.5 (0.1) ^a^
BG0	9.3 (0.1) ^b^	9.3 (0.1) ^b^	9.2 (0.1) ^b^	9.2 (0.0) ^b^	9.2 (0.0) ^b^	9.2 (0.1) ^b^	9.1 (0.1) ^b^	9.1 (0.1) ^b^	9.1 (0.1) ^b^	9.1 (0.1) ^b^	9.1 (0.1) ^b^	9.1 (0.1) ^b^
BG1	9.3 (0.0) ^b^	9.3 (0.0) ^b^	9.3 (0.0) ^b^	9.3 (0.0) ^b^	9.3 (0.0) ^b^	9.3 (0.0) ^b^	9.2 (0.0) ^b^	9.2 (0.0) ^b^	9.1 (0.0) ^b^	9.1 (0.0) ^b^	9.1 (0.0) ^b^	9.1 (0.0) ^b^
BG2	9.4 (0.0) ^b^	9.3 (0.1) ^b^	9.3 (0.0) ^b^	9.3 (0.0) ^b^	9.3 (0.0) ^b^	9.3 (0.0) ^b^	9.2 (0.0) ^b^	9.2 (0.0) ^b^	9.2 (0.0) ^b^	9.2 (0.1) ^b^	9.2 (0.1) ^b^	9.1 (0.1) ^b^
NC	7.8 (0.0) ^c^	7.9 (0.1) ^c^	7.8 (0.1) ^c^	7.9 (0.1) ^c^	8.0 (0.1) ^c^	8.3 (0.3) ^c^	8.4 (0.2) ^c^	8.4 (0.3) ^c^	8.3 (0.2) ^c^	8.2 (0.2) ^c^	8.2 (0.2) ^c^	8.3 (0.2) ^c^

Values indicate mean (standard deviation). F0, F1, and F2: MTA Flow White mixed in three different consistencies—F0 (0.19 g powder per 0.12 g liquid), F1 (0.26 g powder per 0.12 g liquid), and F2 (0.19 g powder per 0.04 g liquid); BG0, BG1, and BG2: Nishika Canal Sealer BG multi mixed in three different consistencies—BG0 (no powder added), BG1 (0.03 g powder per 0.09 g paste), and BG2 (0.06 g powder per 0.09 g paste); NC: negative control. Values with different superscript letters (^a, b, c^) within the same column indicate statistically significant differences between the groups at that specific time point (*p* < 0.05 based on two-way repeated-measures ANOVA with Bonferroni correction).

**Table 6:** Calcium ion release (ppm) from F and BG at different consistencies over 28 days.

Day	3	5	7	10	12	14	17	19	21	24	26	28
F0	470.0 (20.0) ^a^	430.0 (20.0) ^a^	417.5 (20.6) ^a^	392.5 (9.6) ^a^	395.0 (17.3) ^a^	382.5 (28.7) ^a^	370.0 (27.1) ^a^	350.0 (25.8) ^a^	280.0 (29.4) ^a^	257.5 (26.3) ^a^	245.0 (25.2) ^a^	225.0 (12.9) ^a^
F1	470.0 (20.0) ^a^	420.0 (23.1) ^a^	400.0 (24.5) ^a^	377.5 (28.7) ^a^	360.0 (32.7) ^a^	355.0 (25.2) ^a^	342.5 (41.9) ^a^	327.5 (42.7) ^a^	292.5 (51.9) ^a^	265.0 (44.3) ^a^	247.5 (35.9) ^a^	227.5 (22.2) ^a^
F2	500.0 (23.1) ^a^	410.0 (20.0) ^a^	392.5 (33.0) ^a^	392.5 (34.0) ^a^	382.5 (34.0) ^a^	377.5 (36.9) ^a^	370.0 (29.4) ^a^	355.0 (23.8) ^a^	265.0 (59.7) ^a^	252.5 (55.0) ^a^	232.5 (45.7) ^a^	202.5 (33.0) ^a^
BG0	117.5 (5.0) ^b^	107.5 (5.0) ^b^	102.0 (5.4) ^b^	97.8 (4.5) ^b^	96.3 (4.5) ^b^	93.3 (11.6) ^b^	65.5 (14.3) ^b^	75.3 (15.3) ^b^	61.3 (9.8) ^b^	59.5 (10.9) ^b^	59.5 (9.9) ^b^	58.0 (9.5) ^b^
BG1	117.5 (5.0) ^b^	105.0 (5.8) ^b^	89.0 (8.2) ^b^	90.3 (5.3) ^b^	86.5 (6.0) ^b^	83.5 (7.4) ^b^	54.0 (12.1) ^b^	62.0 (8.2) ^b^	48.0 (4.3) ^b^	45.0 (3.5) ^b^	45.8 (2.2) ^b^	43.8 (3.0) ^b^
BG2	125.0 (5.8) ^b^	102.5 (5.0) ^b^	80.5 (8.7) ^b^	81.8 (10.2) ^b^	82.0 (9.6) ^b^	78.0 (9.9) ^b^	53.5 (13.8) ^b^	55.0 (6.8) ^b^	44.0 (3.3) ^b^	43.0 (5.0) ^b^	45.5 (2.5) ^b^	42.5 (2.1) ^b^
NC	0.0 (0.0) ^c^	0.0 (0.0) ^c^	0.0 (0.0) ^c^	0.0 (0.0) ^c^	0.0 (0.0) ^c^	0.0 (0.0) ^c^	0.0 (0.0) ^c^	0.0 (0.0) ^c^	0.0 (0.0) ^c^	0.0 (0.0) ^c^	0.0 (0.0) ^c^	0.0 (0.0) ^c^

Values indicate mean (standard deviation). F0, F1, and F2: MTA Flow White mixed in three different consistencies—F0 (0.19 g powder per 0.12 g liquid), F1 (0.26 g powder per 0.12 g liquid), and F2 (0.19 g powder per 0.04 g liquid); BG0, BG1, and BG2: Nishika Canal Sealer BG multi mixed in three different consistencies—BG0 (no powder added), BG1 (0.03 g powder per 0.09 g paste), and BG2 (0.06 g powder per 0.09 g paste); NC: negative control. Values with different superscript letters (^a, b, c^) within the same column indicate statistically significant differences between the groups at that specific time point (*p* < 0.05 based on two-way repeated-measures ANOVA with Bonferroni correction).

**Table 7:** Mass change (%) and water sorption (%) of F and BG prepared at different consistencies after 28 days of immersion.

Material	Property	1	2	3	4	Mean ± SD
F0	Mass change (%)	−12.8	−11.3	−7.0	−13.1	−11.1 ± 2.8 ^b^
	Water sorption (%)	19.5	21.6	17.6	24.1	20.7 ± 2.8 ^b^
F1	Mass change (%)	−5.1	−3.6	−8.5	−7.2	−6.1 ± 2.2 ^b^
	Water sorption (%)	12.2	9.3	15.2	12.5	12.3 ± 2.4 ^b^
F2	Mass change (%)	19.7	19.2	13.7	12.7	16.3 ± 3.7 ^a^
	Water sorption (%)	5.2	5.3	6.7	4.1	5.3 ± 1.1 ^a^
**Material**	**Property**	**1**	**2**	**3**	**4**	**Mean** **± SD**
BG0	Mass change (%)	−9.5	−9.9	−14.4	−13.2	−11.8 ± 2.4 ^a^
	Water sorption (%)	18.0	18.9	19.8	16.9	18.4 ± 1.2 ^a^
BG1	Mass change (%)	−10.7	−9.7	−6.7	−11.7	−9.7 ± 2.2 ^a^
	Water sorption (%)	16.4	15.8	11.1	14.5	14.5 ± 2.3 ^ab^
BG2	Mass change (%)	−10.5	−13.7	−12.5	−8.5	−11.3 ± 2.3 ^a^
	Water sorption (%)	8.1	5.2	10.6	13.0	9.2 ± 3.4 ^b^

F0, F1, and F2: MTA Flow White mixed in three different consistencies—F0 (0.19 g powder per 0.12 g liquid), F1 (0.26 g powder per 0.12 g liquid), and F2 (0.19 g powder per 0.04 g liquid); BG0, BG1, and BG2: Nishika Canal Sealer BG multi mixed in three different consistencies—BG0 (no powder added), BG1 (0.03 g powder per 0.09 g paste), and BG2 (0.06 g powder per 0.09 g paste). Different lowercase letters (^a, b^) indicate significant differences among the groups within the same property (*p* < 0.05).

## Data Availability

The data presented in this study are available on request from the corresponding author.

## Supplementary Materials

The following supporting information can be downloaded at: https://www.mdpi.com/article/10.3390/jfb17050220/s1. Figure S1: Changes in pH over time across different treatment groups; Figure S2: Changes in calcium ion over time across different treatment groups; Figure S3: Mass change of the tested materials; Figure S4: Time-dependent pH changes of BG at different consistencies over 28 days; Figure S5: Calcium ion release (ppm) from BG at different consistencies over 28 days; Figure S6: Water sorption percentage of F and BG at different consistencies over 28 days; Table S1: PRILE 2021 checklist; Table S2: pH in different samples over a 28-day storage period; Table S3: Concentrations of calcium ion (ppm) in different samples over a 28-day storage period; Table S4: Mass change (%) of the tested materials after 28 days of immersion; Table S5: Time-dependent pH changes of BG at different consistencies over 28 days; Table S6: Calcium ion release (ppm) from BG at different consistencies over 28 days; Table S7: Water sorption (%) of F and BG prepared at different consistencies after 28 days of immersion.

## Abbreviations

The following abbreviations are used in this manuscript:

ANOVA	Analysis of variance
BG	Nishika Canal Sealer BG multi
F	MTA Flow White
HP	MTA Repair HP
hPDLCs	Human periodontal-ligament-derived cells
MTA	Mineral trioxide aggregate
MTT	3-[4,5-dimethylthiazol-2-yl]-2,5 diphenyl tetrazolium bromide
NC	Negative control
PRILE	Preferred Reporting Items for Laboratory Studies in Endodontology
SA/V	Surface area-to-volume
SD	Standard deviation
