# Supplementary material for: Effect of Powder-to-Liquid Ratio on pH, Calcium Ion Release, and Solubility Behaviors of Endodontic Bioceramics: An In Vitro Study
Source: J Funct Biomater. 2026 May 2;17(5):220. doi: 10.3390/jfb17050220 (PMC13207203; doi:10.3390/jfb17050220)
Supplement: Supplementary file 1 [file jfb-17-00220-s001.zip › Figures S1-S6.pdf]

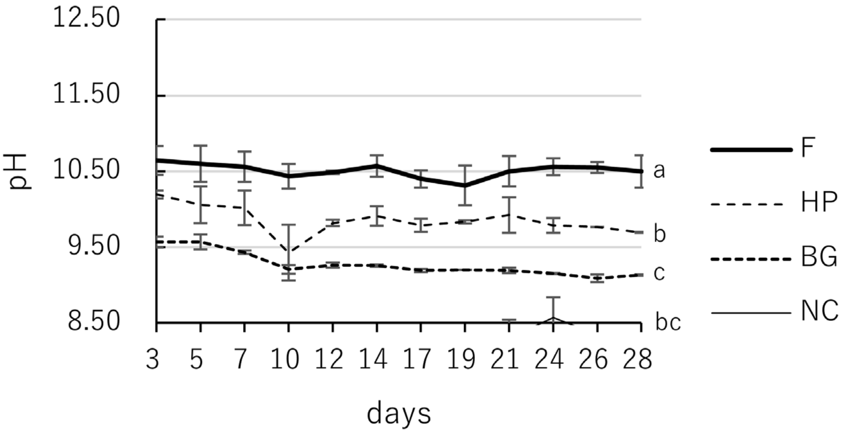

Figure S1. Changes in pH over 28 days across different treatment groups. pH values were measured at specific time points (days 3, 5, 7, 10, 12, 14, 17, 19, 21, 24, 26, and 28). F: MTA Flow White; HP: MTA Repair HP; BG: Nishika Canal Sealer BG multi; NC: negative control. Values indicate mean, and error bars represent standard deviation, and solid, thin solid, dashed, and dotted lines represent different materials as indicated in the legend. Values with different indicator (a, b, c) indicate statistically significant differences between the materials ($p < 0.05$).

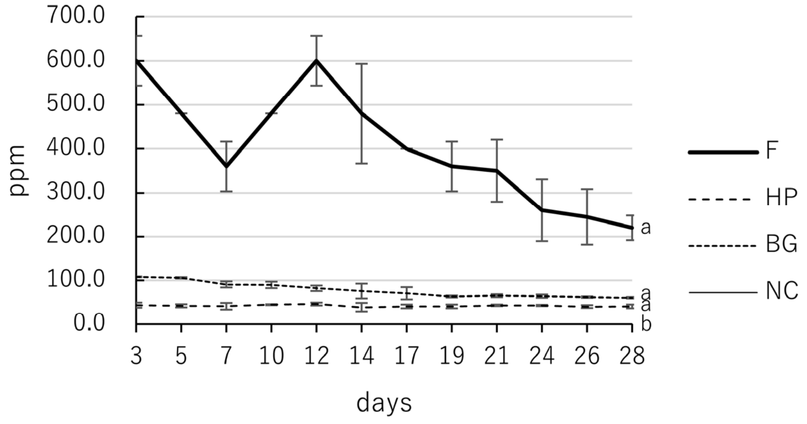

Figure S2. Changes in calcium ion over 28 days across different treatment groups. Calcium ions were measured at specific time points (days 3, 5, 7, 10, 12, 14, 17, 19, 21, 24, 26, and 28). F: MTA Flow White; HP: MTA Repair HP; BG: Nishika Canal Sealer BG multi; NC: negative control. Values indicate mean, and error bars represent standard deviation, and solid, thin solid, dashed, and dotted lines represent different materials as indicated in the legend. Values with different indicator (a, b) indicate statistically significant differences between the materials ($p < 0.05$).

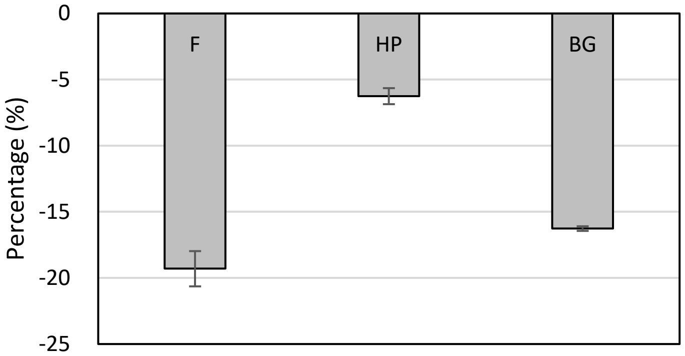

Figure S3. Mass change (%) of the tested materials after 28 days of immersion in deionized water. Bars represent the mean percentage change for F, HP, and BG. F: MTA Flow White; HP: MTA Repair HP; BG: Nishika Canal Sealer BG multi. Bars represent the mean

value with error bars indicating standard deviation. No statistically significant differences were observed between the groups ($p > 0.05$).

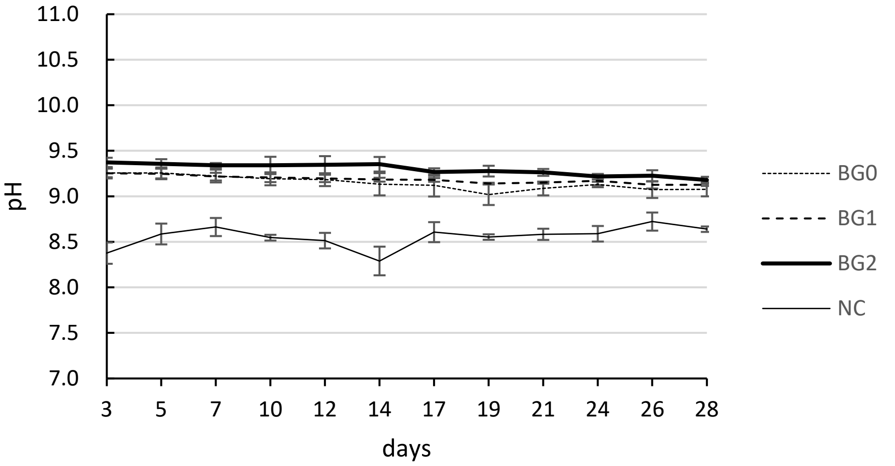

Figure S4. Time-dependent pH changes of Nishika Canal Sealer BG multi (BG) at different consistencies over 28 days. Samples were immersed in deionized water, and the eluate was analyzed on days 3, 5, 7, 10, 12, 14, 17, 19, 21, 24, 26, and 28. Consistencies for BG: BG0 (thin), BG1 (thick), and BG2 (putty). Detailed mixing ratios are provided in the Materials and Methods section; NC represents the negative control. Values indicate mean, and error bars represent standard deviation, and solid, thin solid, dashed, and dotted lines represent BG at different consistencies as indicated in the legend. All tested materials exhibited significantly higher values than the NC group ($p < 0.05$). For specific pairwise comparisons between BG0, BG1, BG2, and NC, see Table S5.

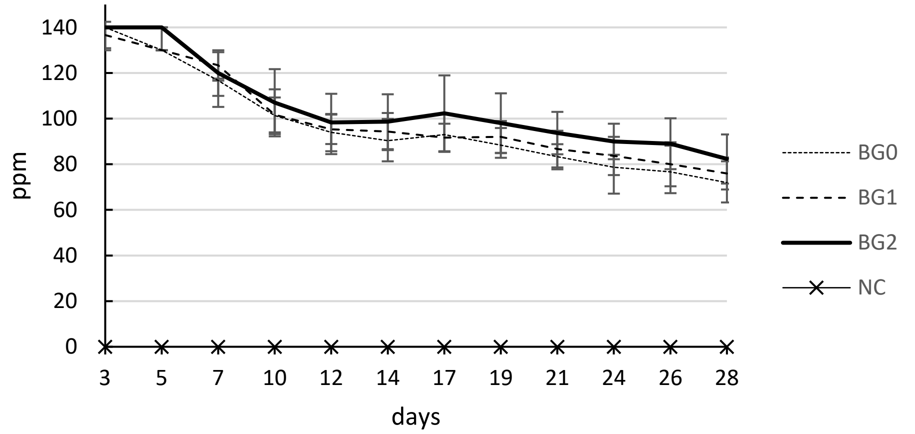

Figure S5. Calcium ion release (ppm) from Nishika Canal Sealer BG multi (BG) at different consistencies over 28 days. Samples were immersed in deionized water, and the eluate was analyzed on days 3, 5, 7, 10, 12, 14, 17, 19, 21, 24, 26, and 28. Consistencies for BG: BG0 (thin), BG1 (thick), and BG2 (putty). Detailed mixing ratios are provided in the Materials and Methods section; NC represents the negative control. Values indicate mean, and error bars represent standard deviation, and solid, thin solid, dashed, and dotted lines represent BG at different consistencies as indicated in the legend. All tested materials exhibited significantly higher values than the NC group ($p < 0.05$). For specific pairwise comparisons between BG0, BG1, and BG2, see Table S**6**.

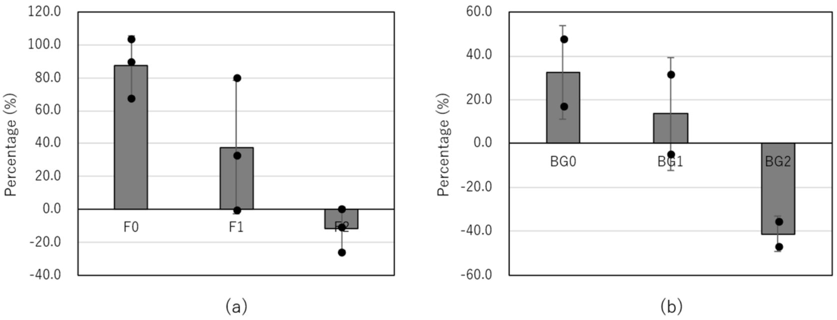

Figure S6. Water sorption percentage of (a) MTA Flow White (F) and (b) Nishika Canal Sealer BG multi (BG) at different consistencies over 28 days of immersion in deionized water. Consistencies for F: F0 (thin), F1 (thick), and F2 (putty); Consistencies for BG: BG0 (thin), BG1 (thick), and BG2 (putty). Detailed mixing ratios are provided in the Materials and Methods section; NC represents the negative control. Individual data points are shown as black circles, and bars represent the mean value with error bars indicating standard deviation. No significant differences among the F groups (p = 0.06) or the BG groups (p = 0.15).
